# Modelling the impact of single vs. dual presentation on visual discrimination across resolutions

**DOI:** 10.1177/17470218241255670

**Published:** 2024-06-19

**Authors:** Luke A French, Jason M Tangen, David K Sewell

**Affiliations:** School of Psychology, The University of Queensland, St. Lucia, Queensland, Australia

**Keywords:** Visual categorisation, diffusion decision model, cognitive modelling, global properties, local features

## Abstract

Visual categorisation relies on our ability to extract useful diagnostic information from complex stimuli. To do this, we can utilise both the “high-level” and “low-level” information in a stimulus; however, the extent to which changes in these properties impact the decision-making process is less clear. We manipulated participants’ access to high-level category features via gradated reductions to image resolution while exploring the impact of access to additional category features through a dual-stimulus presentation when compared with single stimulus presentation. Results showed that while increasing image resolution consistently resulted in better choice performance, no benefit was found for dual presentation over single presentation, despite responses for dual presentation being slower compared with single presentation. Applying the diffusion decision model revealed increases in drift rate as a function of resolution, but no change in drift rate for single versus dual presentation. The increase in response time for dual presentation was instead accounted for by an increase in response caution for dual presentations. These findings suggest that while increasing access to high-level features (via increased resolution) can improve participants’ categorisation performance, increasing access to both high- and low-level features (via an additional stimulus) does not.

## Introduction

Our everyday visual categorisation significantly relies on high-level features, which are the end product of blending various elemental visual components into recognisably complex characteristics. For instance, we identify birds by their wings and beaks and bikes by their wheels and handlebars. These seemingly complex features are in fact composed of more basic low-level visual elements such as the colour or shape of the bird’s wings, the location of its beak, or the line orientations and circular shapes that form a bike’s handlebars and wheels. We then compare these features to our established mental representations of stimuli, allowing us to determine the identity of the target.

Although our everyday categorisation predominantly employs a combination of high- and low-level features (Bar, 2004; Schyns & Oliva, 1994), there are occasions where our reliance shifts more towards low-level features to discern categories, especially when visual information is limited (e.g., when stimuli are viewed briefly or from a distance). Interestingly, this shift transpires despite low-level information carrying less diagnostic value.

Humans have a remarkable capacity to extract useful low-level information from stimuli under restricted viewing conditions. Whether limited by rapid presentation (Bacon-Macé et al., 2005; De la Rosa et al., 2011; Rousselet et al., 2003; Thorpe et al., 1996), visual filters and noise (Collin & McMullen, 2005; De la Rosa et al., 2011; Morrone et al., 1983; Oliva & Schyns, 2000; Schyns & Oliva, 1994; Thompson & Tangen, 2014), or low-resolution images (Bachmann, 1991; Harmon & Julesz, 1973; Torralba, 2009), people often perform surprisingly well when tested for memory of such stimuli (Searston et al., 2019; Wolfe & Kuzmova, 2011) and when categorising stimuli presented in these ways (Searston et al., 2019; Torralba, 2009). This sensitivity to low-level information used during categorisation has been further observed through research that has found low-level stimulus properties can predict, or is associated with, patterns of neural response (Andrews et al., 2010, 2015; Watson et al., 2014).

A category is clearly distinguishable when there’s a recognisable difference between its within-category variance (i.e., the natural variation in features or traits that are common to members of a particular category, like various types of feathers in different bird species) and the between-category variance (i.e., how these shared traits vary when compared with those found in non-category members, such as feathers for birds vs. fur for bats). Hence, while wings can often be an identifying factor common to birds, bats, and planes, it is the unique shape, colour, and function of these wings that serve as critical identifying features.

Discriminability reflects our capacity to distinctly separate these two types of feature variance. Although the features such as shape, colour, or functionality might differ among bird species, the divergence between birds and non-birds is substantially broader. For features that might appear in multiple categories, such as wings, improving discriminability requires sensitivity to the variation in this feature that is unique to different categories (i.e., the differences between a bird’s wings and those of a plane).

**Figure 1.**
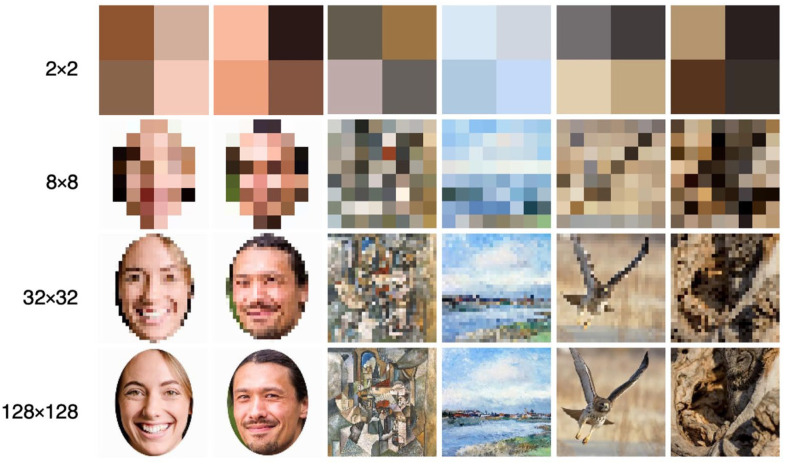
Example of the 2 × 2, 8 × 8, 32 × 32, and 128 × 128 resolution stimuli used in Searston et al. (2019). *Note.* Image categories from left to right: Female, Male, Cubist, Impressionist, Accipitridae, Strigidae.

Searston et al. (2019) conducted two experiments in which they sought to clarify our ability to discriminate low-level between- and within-category features by examining stimuli with progressively lower resolutions. Their methodology involved downsampling a variety of images to lower resolutions through a nearest-neighbour algorithm. They then rescaled all images to 256 × 256 pixels to preserve a consistent presentation size using the same algorithm. Consequently, the resultant images exhibited less pixel variation, providing a more uniform representation of a given image and its category (see Figure 1). Each descending level of resolution invariably led to the loss of more high-level information, leaving mainly low-level attributes intact. Searston et al. (2019) coined the term *style* to specifically characterise such low-level information as “the residual redundant information distributed within and across extremely low-resolution image sets” (p. 574).

Searston et al. (2019) found that, even without any preliminary information about the images or categories, participants could identify previously presented 1 × 1 stimuli and categorise 2 × 2 stimuli, both at above chance levels. Their findings suggested that under such reduced resolutions, participants depend solely on what was described as “stylistic information,” underscoring that this extremely low-level information can suffice for both recognition and categorisation. As expected, however, participants’ ability to discriminate improved as resolutions increased, parallel to the re-emergence of more diagnostic high-level features.

Much like higher-level features, our ability to perform visual categorisation using this low-level information hinges on our sensitivity to the covariance of these features (e.g., colour, shape, contrast, etc.) across multiple instances (Searston et al., 2019). For instance, a bird in flight often produces a distinct “T” silhouette, but this shape could vary depending on the bird species and viewing angle. Similarly, a bat or plane could form a comparable silhouette, making this kind of information less distinctive compared with a combination of easily distinguishable high-level features like a beak, feathers, or wings. When comparing with the more constrained low-level information found in low-resolution stimuli, this consistency of multiple high-level features supporting the same response should serve to provide stronger converging evidence of the most likely identity of the stimulus.

If such a coherence of high-level features helps enhance our categorisation ability, it stands to reason that broadening the availability of relevant low-level features could also bolster categorisation through improved feature coherence. One way to accomplish this is by introducing a second stimulus from the same target category.

Presenting multiple stimuli grants participants access not only to the features within each image but also to the visual information that is shared or distributed across the images. Therefore, if categorisation benefits from this added coherence of low-level feature information across exemplars, providing an extra low-resolution stimulus potentially enhances this. Take, for example, a single low-resolution image that seemingly depicts a bird in flight (e.g., a dark “T” shaped silhouette against a lighter blue background). With the addition of a second image that further support this hypothesis (e.g., a dark “V” silhouette set against a backdrop of lighter blue and brown divided by a horizontal line), the overall evidence favouring the idea of the subject being a bird in flight increases. Likewise, in cases where the information within an image is unusually ambiguous, the inclusion of a second, potentially less ambiguous image should aid in enhancing categorisation irrespective of resolution.

Presenting participants with two stimuli from a single category should emphasise both the crucial similarities and irrelevant differences between instances within that category. This diversity in feature distributions distinguishes our study from others focused on typical redundancy gain effects where a redundant second stimulus can enhance participants’ speed and accuracy (Mordkoff & Yantis, 1991). It is important to note that while each stimulus in a trial belongs to the same category, it is not just the information within each image that is diagnostic. The information across the image pair also plays a crucial role.

Earlier studies have captured our *capacity* to depend on low-level information in decision-making. Our goal is to provide a more detailed assessment of *how* changes to the availability of low-level information affect this process. To do so, we will ask participants to categorise single and dual presentations of the stimuli from Searston et al.’s (2019) study.

Higgins and Ross (2011) have carried out similar work whereby participants were shown either single or dual-stimulus presentations during category learning. They reported that the dual-stimulus presentation did not significantly increase accuracy during learning, or in subsequent tests. However, our methodology differs significantly in key aspects, enabling us to build upon their findings.

First, the earlier study by Higgins and Ross (2011) was based on a same/different task where participants decided whether a presented stimulus belonged to the same category as a known (i.e., labelled) stimulus or to a different category. This known stimulus was either presented just before the target stimulus (single image condition) or concurrently (dual-image condition). Hence, participants could leverage information from two stimuli to make their decision, irrespective of the presentation condition. Contrarily, in our current study, categorisation of single image presentations depends entirely on identifying the category of the presented stimulus rather than comparing it to a pre-identified one. In addition, in the dual-stimulus condition, both images always belong to the same category and the resultant stimuli combinations are consistently either AA or BB, never AB or BA. This draws a decision-level equivalence to stimuli presented in the single image condition (A or B).

However, a significant divergence between our study and that of Higgins and Ross (2011) lies in that they only examined stimuli of a single resolution (the used resolution was not disclosed, but the provided example stimuli appeared clear enough to identify high-level features such as individual feathers and patterns of moss on branches). This limitation restricts any commentary on the contribution of a second image in enhancing discriminability when a participant can only rely on low-level features. Our study aims to determine whether providing individuals with more access to low-level visual information impacts performance similarly to when they have more access to high-level visual information. This particular effect requires an examination of single and dual-stimulus presentations at lower resolutions.

Further delineation in visual information is often made by including “mid-level” features (e.g., texture and depth cues, Groen et al., 2017; spatial envelope, Coggan et al., 2019); however, we do not aim to specify the boundaries of these feature levels. Rather, we view these feature “levels” as a continuum from which there is a clear transition from low to high along with resolution. As image resolution decreases, high-level features are lost at an increased rate compared with low-level features. Although low resolutions such as 2 × 2 can also obscure certain low-level features (e.g., line orientations), some low-level information persists (e.g., colour, contrast) while all high-level information is removed. Discrimination of low-resolution stimuli such as these can only be attributed to reliance on the remaining low-level features, as well as our sensitivity to how these features might vary across members of a category, and between members of differing categories.

To describe how these changes influence decision-making, we will employ the diffusion decision model (DDM) in addition to traditional analysis of discriminability and response time (RT). While conventional analyses based on signal detection theory (SDT; Green & Swets, 1966) often treat choice and RT as distinct variables (see Vandierendonck, 2017, for some notable exceptions), they do offer an effective description and interpretation of experimental outcomes. However, a more comprehensive insight can be gained from an integrated framework, allowing us to thoroughly explore how changes in task performance map onto relevant cognitive processes (Ratcliff et al., 2015). The DDM deconstructs behavioural data into latent psychological constructs that quantitatively gauge the amount of stimulus information the observer can access. This will enable us to measure the comparative evidence across resolutions and as a function of single versus dual presentations. Since each trial for both single and dual presentations requires participants to examine stimuli from a single category (i.e., A or B vs. AA or BB), the decision architecture for both single and dual presentations is considered equivalent. In other words, participants must determine whether overall perceptual evidence, regardless of the number of stimuli presented on a given trial, favours a Category A or Category B response.

**Figure 2.**
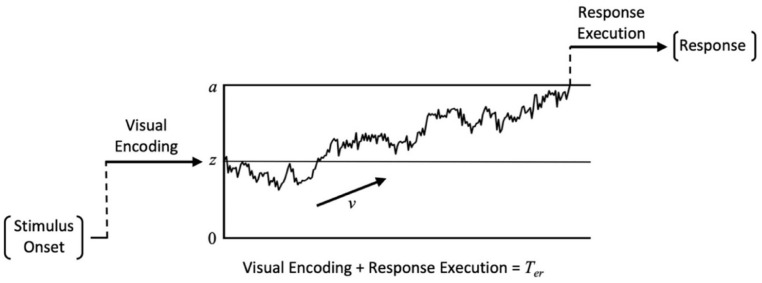
Graphical representation of the diffusion model for a single decision trajectory. *Note.* The mean slope of the jagged accumulation path is characterised by *v*. The irregular nature of the decision trajectory represents the noisy accumulation of conflicting stimulus information. Movement along the *x*-axis represents the passage of time.

The diffusion model (Ratcliff, 1978; Ratcliff & McKoon, 2008) is a model of decision-making, which portrays observers as continually extracting noisy information from a stimulus until they have gathered enough evidence to support a certain response. Figure 2 provides a visual representation of this model.

Evidence accumulation begins from the *start point* (*z*) and moves towards one of two alternate decision boundaries (located at *a* and 0). The distance between these thresholds is the *boundary separation* (*a*) and is indicative of the observer’s response caution. Higher values of *a* indicate a more cautious approach where more evidence is required before committing to a response. The relative distance between the start point and each decision boundary (*a* and 0) represents observer bias, with *z* = *a*/2 corresponding to an unbiased decision. As *z* shifts further away from this halfway point, less evidence is required to reach one of the decision boundaries producing a response bias. To account for between-trial variability, start point is assumed to vary randomly across trials according to a uniform distribution with a mean of *z* and a range of *s*_z._ The rate at which evidence accumulates towards a decision boundary is described by the *drift rate* and reflects the quality and amount of information extracted from the stimulus. High-quality information will produce high drift rates that drive fast RTs and accurate responses while low-quality evidence will produce drift rates closer to 0 that drive slow RTs and near-chance performance. Drift rate is assumed to vary randomly across trials according to a Gaussian distribution with a mean of *v* and standard deviation of *η*. While between-trial variability in evidence accumulation is captured by *η*, each evidence sample is also subject to moment-to-moment noise, as illustrated by the irregular accumulation trajectory in Figure 2. This within-trial variability is characterised by the *diffusion coefficient* (*s*^2^), and is fixed at .01 by convention (Ratcliff, 1978; Ratcliff & McKoon, 2008). While these parameters provide a full description of the time course of the decision process, the model must also account for pre- and post-decision processes involved in stimulus encoding and response execution. The *non-decision time* parameter encompasses the total time spent on these other processes (i.e., visual encoding and response execution). Non-decision time is assumed to vary across trials according to a uniform distribution with a mean of *T*_er_ and a range of *s*_t_.

Diffusion model predictions are generated by attempting to recreate the decision outcomes and RT distribution data of participant data within the constraints of the defined model parameters. There are a number of ways to fit the DDM to data (for a review, see Ratcliff & Childers, 2015). We used the common *G*^2^/chi-square method. The parameter estimates for a given model are determined based on the values that provide the closest approximation of participant data, as assessed by the likelihood ratio statistic *G*^2^ (discussed in more detail below). In fitting the model to participant data and generating model predictions, we can explore which parameters require independent estimates across conditions and, subsequently, identify stimulus-dependent changes to the decision process. For example, when comparing two stimulus conditions, if participants are able to access differing amounts of visual information, we should expect independent estimates of drift rate for each condition, with the higher information condition having a higher drift rate. This distinction between estimated drift rates will allow us to empirically quantify how changing the resolution and quantity of stimuli impact participants’ access to visual information during categorisation.

As a result, in addition to the increases in discriminability across resolution seen by Searston et al. (2019), we expect to find a commensurate increase in drift rate. How resolution affects processing time is less clear with previous studies either not examining RT directly (Searston et al., 2019; Torralba, 2009) or holding presentation time constant as a manipulation (Bachmann, 1991). While increases to drift rate with resolution should reduce RTs, we expect a corresponding increase to non-decision time due to increased encoding costs of more visually complex stimuli, which would serve to increase RT with resolution. We also predict that the addition of a second image will further increase drift rate and discriminability along with RT and non-decision time. We theorise that the addition of a second stimulus will be most beneficial to participants in lower resolutions where individual images have fewer high-level features for participants to draw from. We expect that the second image should help to highlight relevant visual information due to increased availability and coherence of low-level properties across images.

## Method

### Transparency and openness

Our experiment was programmed in Livecode 8.0.0. Raw participant response data were converted to discriminability (*A*) using R (version 4.2.0), the traditional analysis was conducted using SPSS (version 28.0.1.0) and modelling was conducted in MATLAB (2022b). Our sample size, methods, materials, and predicted experimental effects were preregistered on the Open Science Framework and are available online (https://osf.io/5euqm/). Our preregistration identifies that we would be collecting data from 60 participants across four experimental conditions; however, only two of these conditions are included in this publication. Our preregistration does not specify specific models we would be testing but rather highlights an initial focus on examining drift and non-decision time effects with further exploration of other model parameters if supported by our preregistered model selection criteria. Summary data are available online alongside our R and MATLAB scripts (https://osf.io/st59x/).

### Participants

A total of 30 participants (22 females, 8 males) with a mean age of 23.43 (*SD* = 3.31) were recruited through The University of Queensland’s Psychology Research Participation Scheme. Participants received AUD$20/hr for their time and the task took approximately 40 min to complete. We take the approach of Smith and Little (2018) in utilising a small *N* design with a large number of trials relative to the number of participants. In addition to allowing for a more robust measurement of each individual participant, modelling participant data requires sufficient correct and incorrect responses across each level of resolution. By increasing the number of trials for each participant, we can maximise the likelihood of obtaining the required distribution of correct and error responses regardless of task difficulty. Combining across participants, we obtained 2,880 trials per condition. A sensitivity analysis for our sample size determined that the minimum effect size our repeated measures ANOVA could detect with 80% power would be *f* = .32, or η_p_^2^ = .28 for a within-subjects effect, and *f* = .53, or η_p_^2^ = .22 for a between-subjects effect. For a one-tailed single sample comparison to chance performance, with equivalent power, the minimum detectable effect size would be *d* = .46. While this suggests only large effect sizes are likely to be detected by our methodology, we again restate that our methodology is designed primarily to allow us to compare the relative performance of theoretical models generated by the DDM rather than to explore performance solely through significance testing.^1^ Ethics approval was granted by The University of Queensland Health and Behavioural Sciences, Low and Negligible Risk Ethics Sub-Committee (2019000078). Data were collected in 2020.

### Images

The experiment utilised the same three image sets employed by Searston et al. (2019). Each image set, referred to as a “domain” by Searston et al., consisted of images belonging to two categories. The Faces domain included images of males and females, the Paintings domain contained Cubist and Impressionist paintings, and the Birds domain included images of birds from the Accipitridae (e.g., hawks, eagles) and Strigidae (owls) families. All images were resized to a standard size of 256 × 256 pixels using nearest-neighbour interpolation. The Faces domain included a subset of 886 images each of male and female faces originally sourced from the 10k U.S. Adult Faces Database (Bainbridge et al., 2013), while the Painting domain contained 1,296 Cubist and 1,296 Impressionist paintings from an online collection compiled by Searston et al. (2019). The images in the Bird domain included 751 birds from the Strigidae family and 751 from the Accipitridae family, sourced from the Cornell Lab of Ornithology’s (2014) NABirds V1 collection. Although each family contained eight species, the number of instances varied between families. However, within a given species, the number of instances was equal to a species from the alternate family. Refer again to Figure 1 for an example of each stimulus category and resolution.

### Resolution

Searston et al. (2019) modified each image into eight separate resolutions (1 × 1, 2 × 2, 4 × 4, 8 × 8, 16 × 16, 32 × 32, 64 × 64, 128 × 128) using nearest-neighbour interpolation in MATLAB. All images were then upsampled to 256 × 256 pixels to ensure consistent presentation size across all resolution levels. Of these eight resolutions we selected four (2 × 2, 8 × 8, 32 × 32, and 128 × 128) for use in our experiment. We reduced the number of resolutions to increase the number of trials for each remaining resolution, which allowed us to obtain enough data to accurately characterise participant RT distributions within a single testing session. Our main focus was on applying the diffusion model to the data, and this decision was made to optimise the data collected for this purpose.

### Procedure

**Figure 3.**
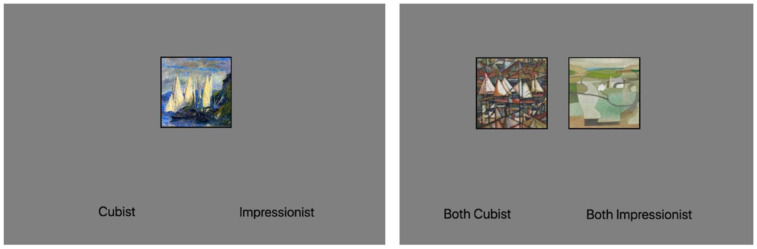
Example trials displaying 128 × 128 resolution paintings for both single and dual presentation conditions.

Participants were randomly assigned into one of two presentation conditions. In the Single Image presentation condition, each trial consisted of a single image centred on the screen presented against a grey background (RGB: [209, 209, 209]). To distinguish the stimulus from the background, a 5-mm black border was added to the edges of the stimulus. In the Dual-Image presentation condition, two images were presented side-by-side 16 mm apart, each offset from the centre by 8 mm. Each of the presented stimuli in the dual-image condition were drawn from the same category (e.g., both owls, both female, etc.) but were separate instances from within that category. Both stimuli in the dual presentation were presented at the same resolution. See Figure 3 for an example trial from both single and dual conditions.

After providing informed consent and viewing a brief instructional video detailing the nature of the resolution manipulation, participants began the experiment (programmed in Livecode 8.0.0). The experiment had three testing blocks with participants permitted to rest between blocks. Each block consisted of a single category domain with the order of blocks counterbalanced across participants. Before beginning each block participants were informed of the categories included in the upcoming trials and provided with an example of each.

During each trial participants were asked to make a category judgement of the stimuli presented on the screen by pressing a key on their keyboard, corresponding to one of two category labels displayed at the bottom of the screen (e.g., “Male,” “Female”). Participants were instructed to respond with the “Z” key to select the category displayed on the bottom left and the “/” key for the category on the bottom right while using their left hand for the “Z” response and their right hand for the “/” response. The location of the category labels and key mapping were consistent for all participants.

For the bird images, the category labels of “Owls” and “Hawks and Eagles” were used in place of their taxonomic labels. For participants in the Dual-Image presentation condition the pre-task instruction video and on-screen labels (e.g., “Both Male” and “Both Female”) clarified that both images belonged to the same category.

On each trial an image from a randomly selected resolution (2 × 2, 8 × 8, 32 × 32, 128 × 128) and within-domain category was presented. The image remained on the screen until the participant made a response, after which a 500-ms blank screen was displayed before the next trial began. No category feedback was provided. For responses faster than 200 ms or slower than 3,000 ms the blank screen was preceded by a “Slow down” or “Speed up” response, presented for 2,000 ms. These prompts were included to encourage participants to respond within a reasonable time frame. The participant’s response and RT were recorded for each trial.

Participants completed 32 trials of each category at each resolution across the three domains for a total of 768 trials. The images used for each participant were randomly selected at the level of resolution, so that a participant would never see the same image at the same resolution. However, it is possible that a participant could encounter the same image at different resolutions, although this was unlikely.

## Results

**Figure 4.**
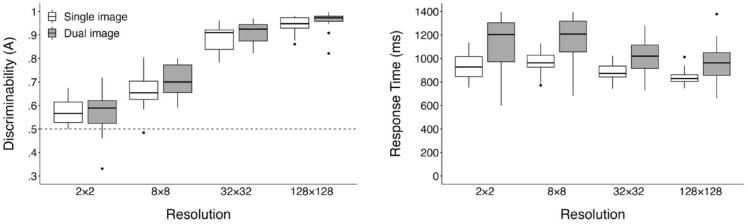
Boxplots displaying mean discriminability (A) and response times (in ms) for each resolution across both single and dual presentations. *Note.* Outliers are *>*1.5 × IQR and are plotted as dots.

While the primary focus of our analysis is the diffusion model results, we first present a more conventional SDT and RT analysis. Responses faster than 200 ms and slower than 3,000 ms were removed from all analyses; this resulted in removal of 403 trials (2% of the total trial count). RT data were not transformed for our analyses to maintain data consistency across traditional and modelling analyses. Participant discriminability was calculated using Zhang and Mueller’s (2005) corrected formula for *Aʹ*, represented as *A*, a non-parametric analogue to *dʹ* where a value of 1 denotes perfect performance and .5 is chance. Our analyses were conducted on participants’ mean scores when collapsing across the three image domains, and RTs include both correct and error responses. Additional exploratory analyses examining differences between performance within each image domain are available online (https://osf.io/st59x/). Participant mean *A* and RTs can be seen in Figure 4.

### ANOVA

We conducted a 2 (stimulus presentation) × 4 (resolution) mixed ANOVA both on participant mean discriminability (*A*) and RTs. Violations of sphericity were addressed by applying the Greenhouse–Geisser correction. Bonferroni adjustments were applied to all follow-up analyses. The upward trend of discriminability shown in the left panel of Figure 4 was substantiated by a significant main effect of image resolution, *F* (2.12, 59.36) = 424.33, *p* < .001, η_p_^2^ = .94. Consecutive pairwise comparisons showed a significant increase in discriminability from 2 × 2 (*M* = .57, *SD* = .07) to 8 × 8 (*M* = .69, *SD* = .08), *t*(29) = 7.6, *p* < .001; 8 × 8 to 32 × 32 (*M* = .90, *SD* = .05), *t*(29) = 24.44, *p* < .001; and 32 × 32 to 128 × 128 (*M* = .95, *SD* = .04), *t*(29) = 9.33, *p* < .001. Replicating the results of Searston et al. (2019), a one-way single sample *t*-test confirmed discriminability for 2 × 2 images to be above chance, *t*(29) = 5.31, *p* < .001, *d* = .97 (*d* as calculated using Cohen’s (1988), original formula].^2^

Contrary to our predictions, the main effect of stimulus presentation on participant discriminability was non-significant, *F*(1, 28) = 1.37, *p* = .252, η_p_^2^ = .05, and we also found a non-significant interaction, *F*(2.12, 59.36) = 1.44, *p* = .245, η_p_^2^ = .05. While these results suggests that the addition of a second category exemplar failed to impact discriminability, caution should be taken in overweighting this non-significant result as our experiment was not designed to provide a comprehensive examination of these effects through significance testing.

As seen in the right panel of Figure 4, the effects of image resolution on participant RTs are less distinct; however, analyses revealed a significant main effect, *F*(1.65, 46.17) = 26.03, *p* = < .001, η_p_^2^ = .48. Pairwise comparisons between adjacent resolution levels revealed a significant decrease in RTs from 8 × 8 (*M* = 1,073.51, *SD* = 222.38) to 32 × 32 (*M* = 964.80, *SD* = 154.81), *t*(29) = 4.46, *p* < .001, and a further decrease from 32 × 32 to 128 × 128 (*M* = 902.44, *SD* = 145.52), *t*(29) = 5.18, *p* < .001.

Examining the main effect of image presentation revealed results more closely aligned with our predictions, as participants were slower in the Dual-Image task (*M* = 1,086.33, *SD* = 193.91) compared with the Single Image (*M* = 903.82, *SD* = 74.22), *F*(1, 28) = 11.59, *p* = .002, η_p_^2^ = .29. The interaction between image resolution and task was non-significant, *F*(1.65, 46.17) = 2.86, *p* = .077, η_p_^2^ = .09. Overall, these results suggest that while discriminability continues to improve with increasing resolution, the benefits in performance speed appear to be limited to higher resolutions.

### DDM

Our main goal was to use the DDM to more thoroughly understand how changes in the availability of high- and low-level visual information impact the decision process during categorisation. To do this, we employed the DDM to clearly isolate individual components of decision-making and how they vary as a function of stimulus properties. By identifying the specific variations (or lack thereof) in model parameters that result in the best-performing model, we can illustrate how these changes affect decision-making. We initially conducted our model analysis on data from the single and dual presentation conditions separately before conducting a combined analysis on the entire dataset. Our motivations for this approach were to fully explore the decision-making processes in both conditions before building towards a more holistic model that examines how these two conditions may differ.

To identify the model parameterisation that best characterised the data, we conducted a series of nested model comparisons. This allowed us to systematically increase model flexibility and weigh any improvement in model fit against the additional degrees of freedom afforded by the more flexible model, striking a balance between fit and complexity. Model fit was optimised utilising SIMPLEX to minimise the likelihood ratio statistic, *G*^2^ defined as:

$$G^{2}=2\sum_{i=1}^{c}n_{i}\sum_{j=1}^{12}p_{ij}\;ln\left(\frac{p_{ij}}{\pi_{ij}}\right)$$

where *c* represents the number of experimental conditions being assessed (four when assessing each presentation condition independently, eight for the combined analysis), and *p* and π each represent the observed and predicted proportion of responses across the response time quantiles (RTQs; 0.1, 0.3, 0.5, 0.7, and 0.9) for both correct and error response distributions. The delineation of five RTQs produces six RT bins with each RTQ demarcating the limits separating each bin, resulting in a maximum value of 12 for the *j* iterator; *n* is set to 192, which corresponds to the number of observations in each condition.

In addition, we computed the Akaike information criterion (AIC) and Bayesian information criterion (BIC) for each model to further assess relative model fit and to ideally provide converging evidence to support a single model. AIC and BIC can be defined as:

$$AIC=\;G^{2}+2k$$

$$BIC=\;G^{2}+k\times \ln(N)$$

where *k* represents the number of parameters and *N* represents total trials across each experiment (768 when fitting each presentation condition independently, 1,536 for the combined fit to data from both presentation conditions).

The below model fits are generated from quantile-averaged group level data. Our model analysis was run on group-averaged data which is treated as data from an “average observer.” RTQs and response rates, for both correct and error responses, are calculated from each participant’s data and then combined into the group average. It is these values that the DDM will attempt to estimate.

**Figure 5.**
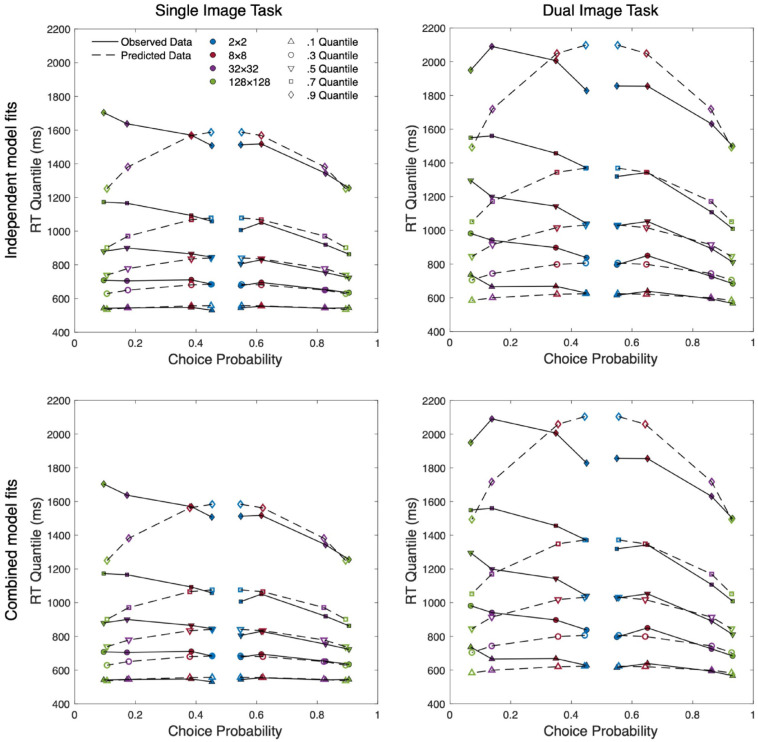
Quantile probability plots of group-averaged data for independent and combined fits of both single presentation and dual presentation data. *Note.* Filled symbols represent observed data. Open symbols represent model predictions. Lines connect adjacent resolutions within each presented quantile for both error (left) and correct (right) data. Independent model fits (top row) present the Standard (4-drift) model and the combined model fits (bottom row) present the Four Drift w/2-a model.

Figure 5 presents four quantile probability plots which display the observed quantile-averaged RT data alongside the model predictions generated by the standard diffusion model. This provides a visual description of how the RT distribution changes across the levels of resolution. For each distribution the 0.1, 0.3, 0.5, 0.7, and 0.9 empirical RTQs are plotted against response probability. Correct and error responses are plotted separately, with data columns on the left-hand side of each plot (choice probability < 0.5) representing the error distributions for each level of resolution and the corresponding correct distributions plotted on the right-hand side (choice probability > 0.5). A choice probability of 0.5 represents chance performance and thus the two innermost marker columns represent the correct (right) and error (left) distributions of the most difficult condition (2 × 2). Moving outwards along the abscissa, and further from the midpoint of the plot, subsequent column pairs plot data for conditions with increasing resolution and decreasing difficulty. Model predictions are shown as open markers and observed data as filled, with each horizontal set representing a quantile. The 0.1 quantile at the bottom of the plot represents the fastest responses (leading edge) of each distribution and the slowest responses (0.9 quantile) are shown at the top of each plot in the figure.

### Model fits

In examining the Single Presentation data we first fit the standard diffusion model, allowing for an independent estimate of drift rate (*v*) in each resolution condition. This model also freely estimates a single value of drift rate variability (*η*), start point variability (*s*_z_), non-decision time (*T*_er_), non-decision time variability (*s*_t_), and boundary separation (*a*). The diffusion coefficient (*s*^2^) was fixed at .01. Start point (*z*) was constrained to *a*/2, representing unbiased responding. Unbiased responding is required as we are modelling correct versus error responses (regardless of stimulus presented): a bias towards being correct or incorrect is theoretically nonsensical.

While this standard model accurately captured the leading edge (see the top-left panel of Figure 5), suggesting a lack of variation in non-decision time across conditions, we chose to formally test whether more visually complex images required differing levels of encoding time. To do this, we contrasted the Standard (4-Drift) model against a Standard + Flexible *T_er_* model, which further allowed independent non-decision time estimates alongside drift. The numerical improvement in fit this second model provided was not sufficient to warrant the increased flexibility, Δ*G*^2^(3) = 2.62, *p* = .454 (see Table 1 for relevant fit indices).

**Table 1.** *G*^2^ and fit indices of each candidate model for the independent model and the combined model analyses.

Model	Single presentation			Dual presentation			Combined analysis		
	*G* ^2^ *(k)*	AIC	BIC	*G* ^2^ *(k)*	AIC	BIC	*G* ^2^ *(k)*	AIC	BIC
**Standard (4*-*Drift)**	**21.49 (9)**	**39.49**	**81.28**	**35.27 (9)**	**53.27**	**95.06**	–	–	–
Standard (4-Drift) + 4-*T_er_*	18.87 (12)	42.87	98.60	31.76 (12)	55.76	111.49	–	–	–
Standard (4-Drift) + 2*-T_er_*	19.64 (10)	39.64	86.08	33.66 (10)	53.66	100.10	–	–	–
Standard (8-Drift)	**–**	–	–	–	–	–	121.00 (13)	147.00	216.38
Standard (8-Drift) w/ 2-*a*	–	–	–	–	–	–	56.80 (14)	84.80	159.52
**Four Drift w/ 2-*a***	–	–	–	–	–	–	**56.88 (10)**	**76.88**	**130.25**
Four Drift w/ 2-*a* & 2-*T_er_*	–	–	–	–	–	–	56.77 (11)	78.77	137.47

*Note. k* denotes number of estimated parameters. Values in italics denote selected model based of each relevant criteria (lowest value for AIC & BIC, lowest significant Δ*G*^2^ for *G*^2^; see text below for details). Selected models (and associated statistics) are bolded.

Due to the large increase in discriminability from 8 × 8 to 32 × 32, and the visually apparent jump in visual information between these resolutions, we also explored a variation of the standard model with two non-decision time estimates. This model held one estimate of non-decision time constant across the lower resolutions (2 × 2 and 8 × 8) and another across the higher resolutions; however, it was outperformed by the Standard (4-Drift) model, Δ*G*^2^(1) = 1.86, *p* = .173.

As shown in the top-left panel of Figure 5, the Standard (4-Drift) model generally aligns with the observed data for correct responses and low-resolution incorrect responses, but there are some notable discrepancies in the model fit for high-resolution errors, particularly in the tails. The model underestimates the variability of the RT distributions for high-resolution errors and overestimates the tails of low-resolution responses (correct and error). However, the model has accurately captured response accuracy across all conditions. QPPs for our rejected models can be found in the supplementary materials. The relevant parameter estimates of our selected models can be found in Table 2.

**Table 2.** Estimated parameter values of the best fitting model for each dataset.

Experiment	*v1*	*v2*	*v3*	*v4*	*a*	*a1*	*a2*	*T_er_*	*η*	*s*_z_	*s*_t_
Single	0.013	0.031	0.102	0.141	0.152	–	–	0.406	2.20e^-10^	1.03e^-10^	1.14e^-10^
Dual	0.012	0.034	0.100	0.141	0.181	–	–	0.409	1.06e^-09^	4.00e-^10^	1.17e^-10^
Combined	0.012	0.033	0.101	0.141	–	0.151	0.182	0.400	3.80e^-09^	5.98e^-09^	1.30e^-10^

*Note*. Model parameters represented by *v* (drift rate), *a* (boundary separation), *T_er_* (non-decision time), *η* (drift rate variability), *s*_z_ (start point variability) and *s*_t_ (non-decision time variability). Increasing numerical notation of *v* represents increasing resolutions. *a1* and *a2* denote estimates for Single and Dual datasets, respectively.

The empirical RT error distributions for high-resolution images are drawn from the smallest response sample with *M* = 9.07 error responses in the 128 × 128, and *M* = 16.37 in the 32 × 32. The five RTQs generated from these samples are therefore subject to greater sampling error (e.g., inattentive responses) than those of higher resolutions (*M* = 36.4 errors for 8 × 8 and *M* = 42.87 errors for 2 × 2). We attribute the relatively poor performance of the model to capture the RT distribution data in these conditions to the lower level of precision in the data.

When independently examining the Dual presentation data, three participants’ RTQs for error responses were not able to be calculated in the 128 × 128 condition as they made fewer than five errors. In cases where this occurred, a participant’s error RTs for the 128 × 128 condition were omitted from the calculation of the group-averaged RT distribution data; however, the remainder of their data (including error response rate) were included.

As with the Single Presentation task, we began by applying the Standard (4-Drift) model and similarly found model predictions to predominantly align with observed data (see top-right panel of Figure 5). Like the previous analysis, the model performed most poorly in estimating the error distributions of the two highest resolution conditions with especially notable discrepancies across the tails of all error and low-resolution correct responses. Again, we compared this standard model with the Standard + Flexible *T_er_* Model, Δ*G*^2^(3) = 3.51, *p* = .315, and found limited improvement in model fit. Finally, we tested the Standard + Dual *T_er_* (high vs. low), which was also outperformed by the standard model, Δ*G*^2^(1) = 1.61, *p* = .204. Relevant parameter estimates can again be seen in Table 2, while QPPs of rejected model can again be found in the supplementary materials.

Our final model analyses examined data from both the single and dual presentation conditions simultaneously to more closely examine the underlying processes that differentiate them. To make these comparisons we simply introduce image presentation as a new condition and require the model to generate predictions for both datasets simultaneously. As with other conditions, if the selected model requires only a single parameter estimate to account for both presentation modes (Single and Dual) we can conclude that the decision component in question is unchanged by the difference in task properties. Alternatively, if independent parameter estimates are required, this suggests differences in the underlying processes.

We began by fitting a standard diffusion model to the data (Standard [8-Drift] model, see Table 1 for model fits). As we have eight conditions across the combined dataset, this constitutes eight independent estimates of drift rate with a single freely estimated value for *a* (boundary separation), *T_er_* (non-decision time), *η* (drift rate variability), *s*_z_ (start point variability), and *s*_t_ (non-decision time variability). The diffusion coefficient (*s*^2^) and start point (*z*) were again fixed at .01 and *a*/2, respectively.

As we noted a marked difference in the shape and range of the RT distributions between experiments (i.e., the dual presentation has a much greater spread across the RT axis), we sought to quantify this change. This variation can be accounted for most readily by changes in boundary separation (*a*) as larger values can elongate the distance between RTQs. To this end, we first fit a more flexible Standard (8-Drift) w/ 2-*a* model produced a significant improvement to model fit, Δ*G*^2^(1) = 64.2, *p* < .001, over the Standard (8-Drift) model suggesting a change in response caution across single and dual presentation tasks, with increased caution seen in the dual task (see Table 2).

To directly test whether providing a second image improved the availability of useful visual information (both high-level and low-level), and reduce model complexity, we fit a model which produced independent drift estimates for each level of resolution but held these values constant across each experiment. If the best-performing model required independent drift rates across both experiments, this would suggest differences in the rate of evidence accumulation between the single and dual presentations. However, the Four Drift w/ 2-*a* model resulted in a non-significant reduction in model fit, Δ*G*^2^(4) = 0.08, *p* = .999, supporting retention of the simpler Four Drift model and suggesting limited differences in drift rates across experiments (see bottom panels of Figure 5.).

Noting the numerically slower .1 quantile in the dual presentation, we fit a model with additional flexibility for non-decision time as *T_er_* can produce a shift in the entire distribution along the RT axis, though without changing its overall shape (unlike *a*). This Four Drift w/ 2-*a* & 2-*T_er_* model which allowed for independent estimates of non-decision time for each experiment did not provide sufficient improvement to model fit to warrant its inclusion as an additional parameter, Δ*G*^2^(1) = 0.11, *p* = .740. QPPs for our rejected models can be found in the supplementary materials. The relevant parameter estimates of our selected models can be found in Table 2.

### Discussion

As expected, participant discriminability data mirrored the results of Torralba (2009) and Searston et al. (2019). Furthermore, drift rates clearly mapped onto choice performance with the selected model for both the independent and combined models requiring resolution-dependent drift rates which increased along with discriminability. The marked jump in discriminability from 8 × 8 to 32 × 32 in both the single and dual presentation tasks is also reflected in drift rates. This considerable increase in discriminability and drift rate seems to suggest a substantial increase in the strength of the available evidence. This change may reflect a transition from a categorisation process predominantly driven by evidence carried by low-level visual features to one that can take advantage of the emergence of specific high-level features that are more informative. This interpretation is consistent with our own experience of the stimuli, where at 32 × 32 we were able to more reliably identify specific image features such as beaks and jewellery.

Changes in RT as a function of resolution were less consistent. Figure 4 shows a downward trend in RT from 8 × 8 onwards, but it is interesting to note that the most striking reduction in RT from 8 × 8 to 32 × 32 is accompanied by the largest increase in discriminability and drift rate. This suggests that a change in the contribution of high-level features to performance occurred, despite no changes in non-decision time. Our motivation for testing of the dual non-decision time models was to ensure the complexity penalties imposed by AIC and BIC were not masking a non-decision time effect in the Standard + Flexible *T_er_* model that more closely captured this change from a low-level to a high-level features-based decision process.

The DDM can account for variation in choice and RT distribution data through boundary separation, non-decision time, and drift rate; however, the selected models can fully account for changes in image resolution through drift rate effects alone. It was originally predicted that the DDM would require resolution-dependent estimates of non-decision time to account for the increasing visual complexity of higher resolutions. Variations in non-decision time are typically attributed to pre-decision processes (i.e., visual encoding) as post-decision processes (i.e., response execution) have no reason to differ across conditions. Although higher resolutions appear to involve an increase in visual information and complexity, our results do not provide evidence of an encoding cost. This is further supported by the largely flat leading edges in Figure 5; if our experiment had produced a detectable encoding cost, we would expect to see slower responses in the 0.1 quantile of higher resolution conditions (e.g., Ratcliff & Smith, 2010; Smith et al., 2014).

In considering the impact of stimulus number, it is clear that while the addition of a second image comes at a cost to participant RT, the combination of the non-significant change to discriminability and the single estimates of drift rate across presentation number strongly suggest a second image does not appear to provide further improvement to participant choice performance. While the increased time spent on the dual-image task implies that the second image is being attended to in some form, having access to the additional information contained in the second image has no discernible effect on participants’ capacity to extract additional diagnostic information to identify the image category.

These findings suggest that people are more efficient at processing additional visual information when it is presented in the form of increased resolution, rather than as an additional image. This is especially striking considering that increases in resolution consistently result in higher choice performance, but adding an additional image does not. These results suggest that people may struggle to effectively process and use the additional information provided by an extra image, despite taking longer to respond. This finding appears to indicate that improvements in choice performance are driven mainly by increasing access to high-level features (via resolution) than to low-level properties (via an additional image).

## General discussion

Our analysis yielded two surprising findings: first, we found that presenting a second category exemplar did not improve the ability to discriminate between categories. Second, our data suggest that the increased RT observed in dual-image presentations is due to increased response caution rather than increased encoding time. The lack of a discrimination effect between participants and the absence of experiment-dependent drift rates show that no improvement in choice performance results from presenting an additional exemplar for categorisation across all image resolutions. This result contrasts with the study by Higgins and Ross (2011). However, in their study, stimuli were presented one after another rather than concurrently, which may account for the different findings. Our study directly probes the effect of providing a second image on visual decision-making processes and demonstrated that an additional image does not necessarily aid in category discrimination.

We have identified three potential reasons for the lack of enhancement when a second stimulus is presented: lack of attention to the second image, insufficient additional helpful information from the second image, or an averaging of the evidence from the two images in the dual task.

Although we did not identify a visual encoding effect across single and dual presentations (as evidenced by having only a single estimated non-decision time), it is unlikely that the second image was entirely ignored. This is suggested by the observed RT and caution effects (i.e., independent estimates of boundary separation across tasks for the combined model), which indicate that participants were, at the very least, aware of the second image’s presence. If participants were entirely indifferent to the second image, these effects would not have surfaced. It would be difficult to argue that participants could selectively rely on the more informative image while discounting the other without enhancing choice performance, as this should result in improved discrimination due to statistical facilitation. Thus, it seems unlikely that participants in the dual condition would deliberately neglect the second image while also increasing their boundary separation, especially considering that response caution is typically under each individual’s control, making such an approach counterproductive.

Another possibility is that the second exemplar did not provide enough additional evidence to reliably enhance discriminability. In principle, a second exemplar should offer participants additional opportunities to evaluate available information, thus providing more supportive evidence for the correct decision. We anticipated this effect to be particularly beneficial for low-resolution images where each image’s available information is limited, maximising the potential advantages of a second information source. However, no benefit for the second image was observed across the entire performance range, which makes this account less plausible. Potentially, the second stimulus in our experiment did not consistently provide category information (both high-level and low-level) that was not already contained in the first stimulus, thus diminishing the second exemplar’s usefulness. In essence, the first stimulus may have provided all necessary low- and high-level feature information required for a category decision, whereas the second stimulus did not add any additional distinctive information.

Another possibility is that participants in the dual condition were responding to the average evidence across both images, rather than the total evidence across the images. We had originally expected that participants in the dual condition would show improved discriminability and higher drift rates because the combined evidence from the two exemplars would increase the overall evidence in support of the correct decision. For example, two images that both somewhat resemble an owl would be stronger evidence than one image that somewhat resembles an owl. We would have expected participants to rely more heavily on the more informative stimulus in cases where one of the presented images was ambiguous and the other was less so. However, from a holistic perspective, if the evidence from each stimulus is given equal weight, information that may be viewed as supportive of a given response when considered in isolation may be seen as less informative when evaluated alongside a second, more ambiguous stimulus. This would result in an overall evidence value for each trial that is the average of the evidence from both images. As a result, we may observe the same mean discriminability and drift rates for both single and dual presentations.

Our results suggest that the low-level information distributed across each within-trial pairing did not significantly improve participant discriminability beyond presenting a single image, regardless of which of the above explanations (or combination thereof) may be true. While this does not completely rule out the possibility that variations in the amount of low-level information can affect the evidentiary value of visual information, it does suggest that a single additional image had only a limited impact in our paradigm. As coined by Searston et al. (2019), style is a kind of low-level information that refers to the distribution of visual similarities shared across category exemplars that covary. While a second source of stylistic/low-level information may highlight some of these similarities, each additional exemplar would further reveal these resemblances. It is not beyond reason that presenting many low-resolution images simultaneously would lead to improved discriminability and increased drift rate compared with presenting a single image.

In contrast to the lack of an effect on discriminability, the increase in participant RTs in the dual condition was as expected. However, while we had anticipated that this change was due to increases in encoding time (reflected in the DDM’s non-decision time parameter), the diffusion model analysis suggests that a different cognitive mechanism may be responsible for this effect. As with increases in resolution, it was predicted that having more visual information available for processing would increase the time required for visual encoding, leading to larger estimates of non-decision time. However, our findings suggest that increases to the quantity of visual information from both resolution and stimulus quantity do not increase non-decision time. Furthermore, the only model parameter that varied across single- and dual-stimulus presentation was boundary separation, which was larger in the dual-stimulus task. This suggests that participants were more cautious in their decision-making in the dual-image task compared with the single.

Despite this increase to the quantity of evidence participants require to make a decision, there was no corresponding improvement in participants’ choice performance. The change in boundary separation required by the model is sufficient to account for the increased RT and improve overall model fit, but insufficient to improve decision outcomes. This appears to suggest an inefficiency in processing information from multiple sources as participants in the single image condition perform equally as well but do so significantly faster. Previous research has found people generally tend to be overcautious when performing simple decision-making tasks (Evans & Brown, 2017), and this effect appears to be evident in the dual-image task.

While our findings suggest that there may not be a significant encoding cost for larger quantities of information from either changes in resolution or the number of images, we do not believe it is accurate to claim that there is truly no encoding cost. Instead, these results may indicate the efficiency with which visual encoding occurs. Previous studies have suggested around 40 ms is sufficient exposure for participants to identify the presence of an animal in novel images (Bacon-Macé et al., 2005). This lack of a detectable encoding effect is unlikely to be entirely due to a failure of the DDM for two reasons: first that past research has shown the DDM is capable of detecting small non-decision time effects (Ratcliff & Smith, 2010), and second that the leading edge across resolutions is inexplicably flat and well captured by the model. It would appear that the previously noted delays to evidence accumulation witnessed across task difficulty are not present in our experimental paradigm.

## Conclusion

In this study, we examined the considerable role that low-level information plays in our visual categorisations under constrained viewing conditions. We not only demonstrated our capacity to categorise low-resolution images, but also examined whether introducing a second exemplar could enhance our ability to correctly categorise visual stimuli. Our findings reveal no discernible benefit to providing an additional visual reference, regardless of whether the stimuli contain predominantly low-level information (low-resolution) or both high- and low-level information (high-resolution). This signals a lack of significant contribution from stylistic, or low-level, information distributed across multiple exemplars. Apart from observing no improvement in discriminability and drift rate, we found that adding a second category exemplar coincided with longer participant RTs. Upon review, these results suggest participants exercised increased response caution rather than incurring a visual encoding cost. A notable finding of this study is the surge in drift rate as image resolution climbs from 8 × 8 to 32 × 32, indicating a significant expansion in available evidence. This underscores the crucial emergence of high-level features and their pivotal contribution to decision-making processes, affirming the intricate balance between high- and low-level visual information in our everyday categorisation tasks.

## Supplemental Material

sj-pdf-1-qjp-10.1177_17470218241255670 – Supplemental material for Modelling the impact of single vs. dual presentation on visual discrimination across resolutions Supplemental material, sj-pdf-1-qjp-10.1177_17470218241255670 for Modelling the impact of single vs. dual presentation on visual discrimination across resolutions by Luke A French, Jason M Tangen and David K Sewell in Quarterly Journal of Experimental Psychology

## Notes

1. It should also be noted that a sensitivity analysis does not take into account the number of trials per cell, a key component of small *N* designs.

2. This result was also consistent when examining Single, *t*(14) = 5.58, *p* < .001, *d* = 1.44, and Dual, *t*(14) = 2.86, *p* = .013, *d* = .74, presentations separately.
