# Supplementary material for: Modelling the impact of single vs. dual presentation on visual discrimination across resolutions
Source: Q J Exp Psychol (Hove). 2024 Jun 19;78(4):827–41. doi: 10.1177/17470218241255670 (PMC11905324; doi:10.1177/17470218241255670)

Supplementary materials

Below we provide the quantile probability plots generated by the alternate models tested during our analyses. Each lefthand panel depicts observed and predicted data for models of the single image presentation condition while those on the right depict the dual image presentation condition. The four plots presented on this page were generated from the independent analyses of single and dual presentations. These were outperformed by the more parsimonious Standard (4-Drift) model. The legend provided in the first plot can be applied to all presented QPPs.

Standard (4-Drift) + 4-T_{er}

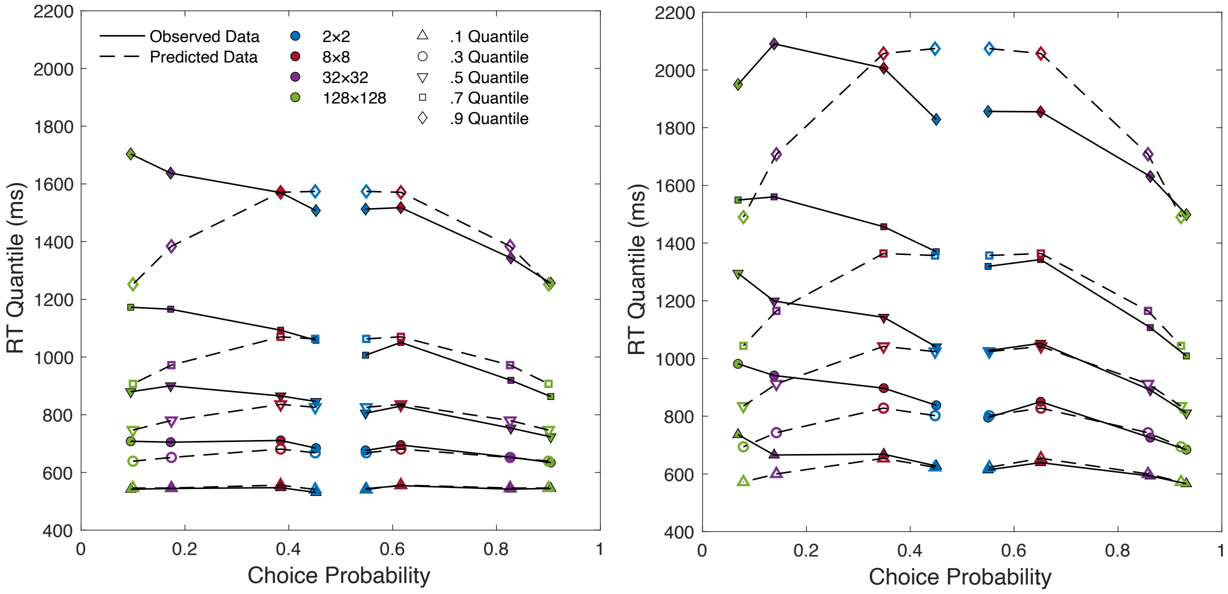

Standard (4-Drift) + 2-T_{er}

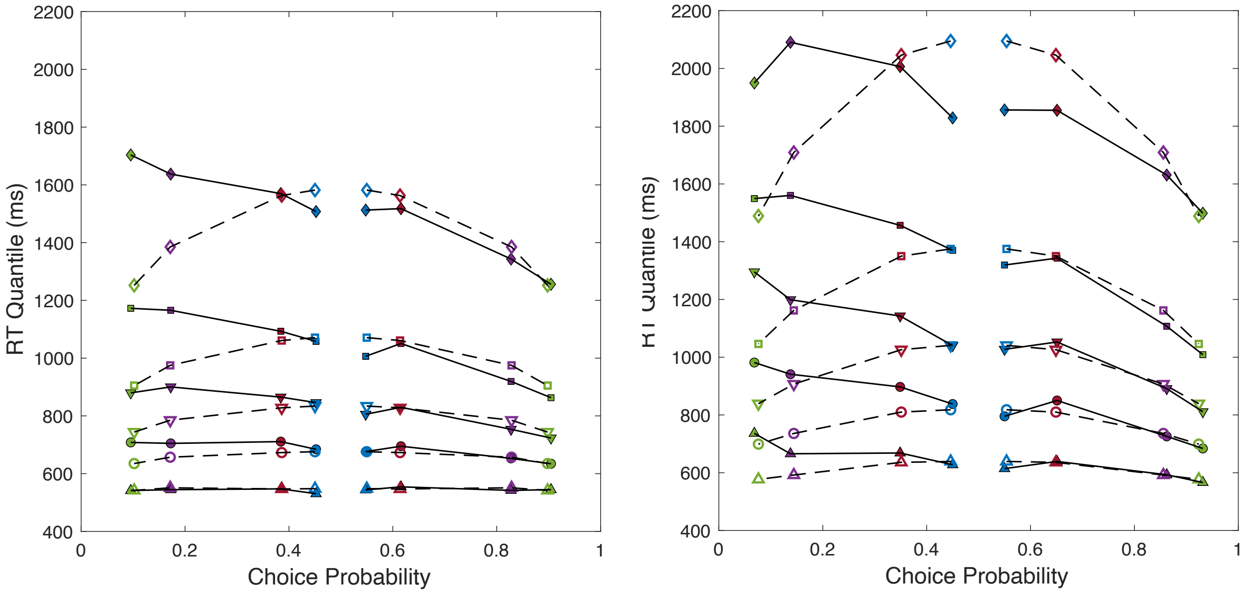

The following six plots were generated from the combined analyses of both single and dual image presentation data. These models were outperformed by the Four Drift w/ 2-*a* model.

Standard (8-Drift)

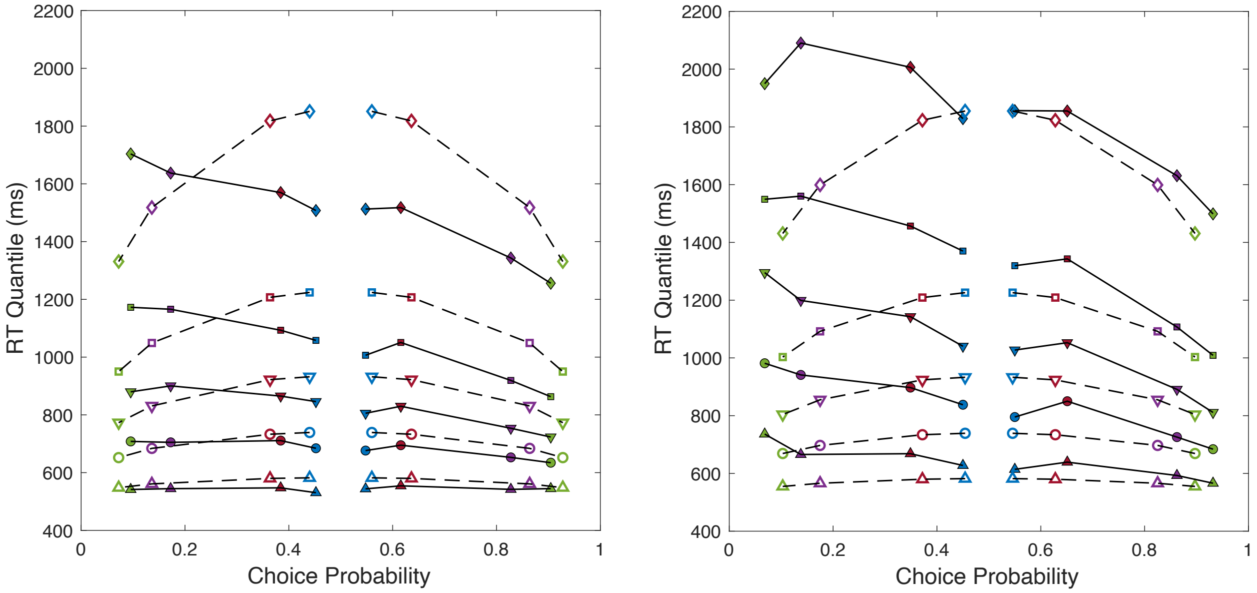

Standard (8-Drift) w/ 2-*a*

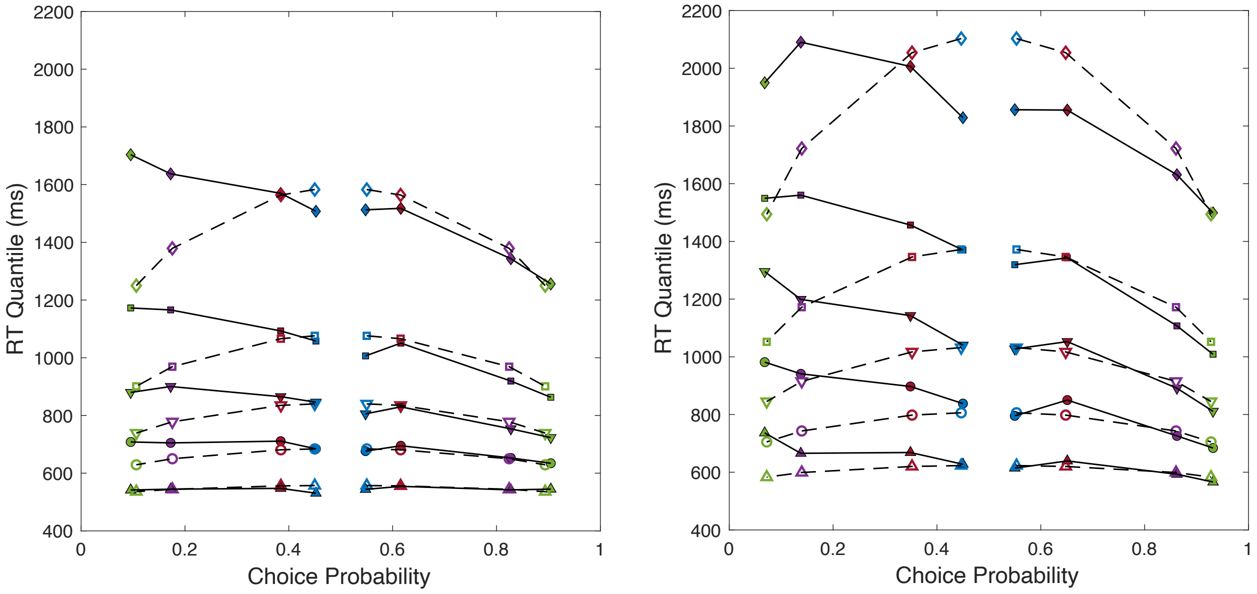

Four Drift w/ 2-a & 2-T_{er}

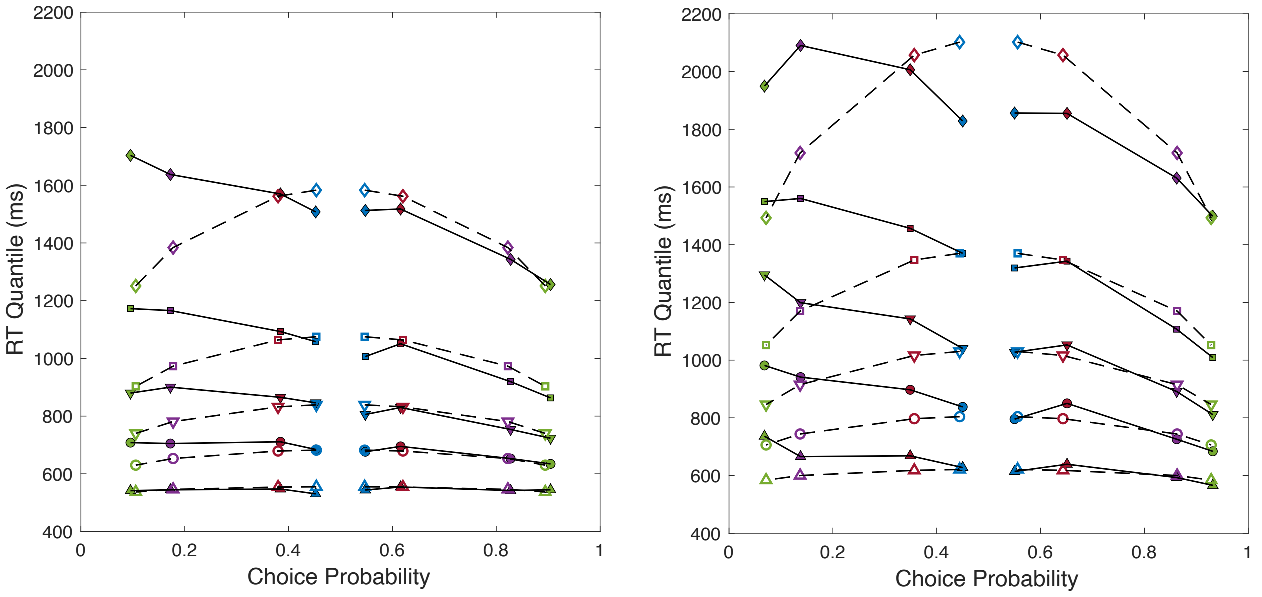

Supplement: sj-pdf-1-qjp-10.1177_17470218241255670 – Supplemental material for Modelling the impact of single vs. dual presentation on visual discrimination across resolutions [file sj-pdf-1-qjp-10.1177_17470218241255670.pdf]
